# Binding and Oligomerization of Modified and Native Bt Toxins in Resistant and Susceptible Pink Bollworm

**DOI:** 10.1371/journal.pone.0144086

**Published:** 2015-12-03

**Authors:** Josue Ocelotl, Jorge Sánchez, Raquel Arroyo, Blanca I. García-Gómez, Isabel Gómez, Gopalan C. Unnithan, Bruce E. Tabashnik, Alejandra Bravo, Mario Soberón

**Affiliations:** 1 Instituto de Biotecnología, Universidad Nacional Autónoma de México, Apdo. Postal 510–3, Cuernavaca, Mor. 62210, (Mexico); 2 Department of Entomology, University of Arizona, Tucson, Arizona, 85721, United States of America; Institute of Vegetables and Flowers, Chinese Academy of Agricultural Science, CHINA

## Abstract

Insecticidal proteins from *Bacillus thuringiensis* (Bt) are used extensively in sprays and transgenic crops for pest control, but their efficacy is reduced when pests evolve resistance. Better understanding of the mode of action of Bt toxins and the mechanisms of insect resistance is needed to enhance the durability of these important alternatives to conventional insecticides. Mode of action models agree that binding of Bt toxins to midgut proteins such as cadherin is essential for toxicity, but some details remain unresolved, such as the role of toxin oligomers. In this study, we evaluated how Bt toxin Cry1Ac and its genetically engineered counterpart Cry1AcMod interact with brush border membrane vesicles (BBMV) from resistant and susceptible larvae of *Pectinophora gossypiella* (pink bollworm), a global pest of cotton. Compared with Cry1Ac, Cry1AcMod lacks 56 amino acids at the amino-terminus including helix α-1; previous work showed that Cry1AcMod formed oligomers *in vitro* without cadherin and killed *P*. *gossypiella* larvae harboring cadherin mutations linked with >1000-fold resistance to Cry1Ac. Here we found that resistance to Cry1Ac was associated with reduced oligomer formation and insertion. In contrast, Cry1AcMod formed oligomers in BBMV from resistant larvae. These results confirm the role of cadherin in oligomerization of Cry1Ac in susceptible larvae and imply that forming oligomers without cadherin promotes toxicity of Cry1AcMod against resistant *P*. *gossypiella* larvae that have cadherin mutations.

## Introduction

The widespread bacterium *Bacillus thuringiensis* (Bt) produces insecticidal crystalline (Cry) proteins that have been used in sprays for pest control for more than 50 years [1]. In addition, transgenic crops that produce Bt proteins to kill pests were first commercialized 20 years ago and were planted on 78 million hectares worldwide in 2014 [2, 3]. Cry toxins are effective against their target insect pests, but are not toxic to plants, vertebrates, or most non-target invertebrates [2, 4, 5, 6]. The evolution of resistance in insect pests is the primary threat to the long-term efficacy of Bt toxins. Resistance has evolved in the laboratory, and in the field where Bt toxins were used in sprays or transgenic crops [7, 8, 9, 10, 11, 12]. Reduced binding of Bt toxins to larval midgut membranes is the most common and most potent mechanism of resistance [13, 14].

Although models of the mode of action of Bt toxins differ in some ways, all agree that toxicity of Cry1A proteins requires ingestion, solubilization in the midgut, and binding to larval midgut proteins, such as alkaline phosphatase (ALP), aminopeptidase N (APN), and cadherin [15, 16, 17, 18]. In the pore formation model, binding of toxin leads to formation of non-selective pores in midgut cell membranes and cell lysis [15]. Alternatively, the signaling model proposes that binding of toxin to cadherin initiates a magnesium-dependent signaling pathway that kills cells [19]. Recent reviews conclude that experimental support is stronger for the pore formation model than the signaling model, yet some details of the pore formation model remain unresolved [20, 21].

According to a variant of the pore formation model called the sequential binding model, binding to cadherin facilitates proteolytic removal of helix α-1 from the amino-terminus of Cry1A toxins, eventually triggering toxin oligomerization and irreversible binding caused by insertion of oligomers into cell membranes [16–18, 22, 23]. However, the importance of oligomerization has been questioned [20, 21]. All previous evidence showing an association between oligomerization and toxicity comes from testing susceptible insect strains [24, 25].

In this study, to better understand Bt mode of action and mechanisms of resistance, we evaluated how Bt toxin Cry1Ac and its genetically engineered counterpart Cry1AcMod interact with brush border membrane vesicles (BBMV) from resistant and susceptible larvae of *Pectinophora gossypiella* (pink bollworm), a global pest of cotton. Resistance to Cry1Ac is linked with mutations that disrupt a Cry1Ac-binding cadherin protein in three major lepidopteran pests, including *P*. *gossypiella* [26, 27, 28]. The AZP-R strain of *P*. *gossypiella* harbors three recessive cadherin mutations that confer >1000-fold resistance to Cry1Ac [27, 29–31]. However, AZP-R and some resistant strains of several other species remain relatively susceptible to the genetically modified toxins Cry1AbMod and Cry1AcMod [31, 32]. The Cry1AMod protoxins have a deletion of 56 amino acids at their amino-terminus including helix α-1, and do not require cadherin to form toxin oligomers *in vitro* [31]. The role of cadherin in toxin oligomerization has been documented *in vitro* with toxin-binding cadherin fragments from susceptible larvae [23]. However, previous work has not determined if cadherin mutations in resistant insects interfere with oligomerization. Here we compared the resistant strain AZP-R with a susceptible strain in terms of cadherin expression, as well as binding and oligomerization of Cry1Ac and Cry1AcMod. The results imply that the absence of wild type cadherin in AZP-R blocks oligomerization of Cry1Ac, but not Cry1AcMod, confirming that oligomerization is a key step in the toxic pathway.

## Results

### Cadherin Expression

**Fig 1:**
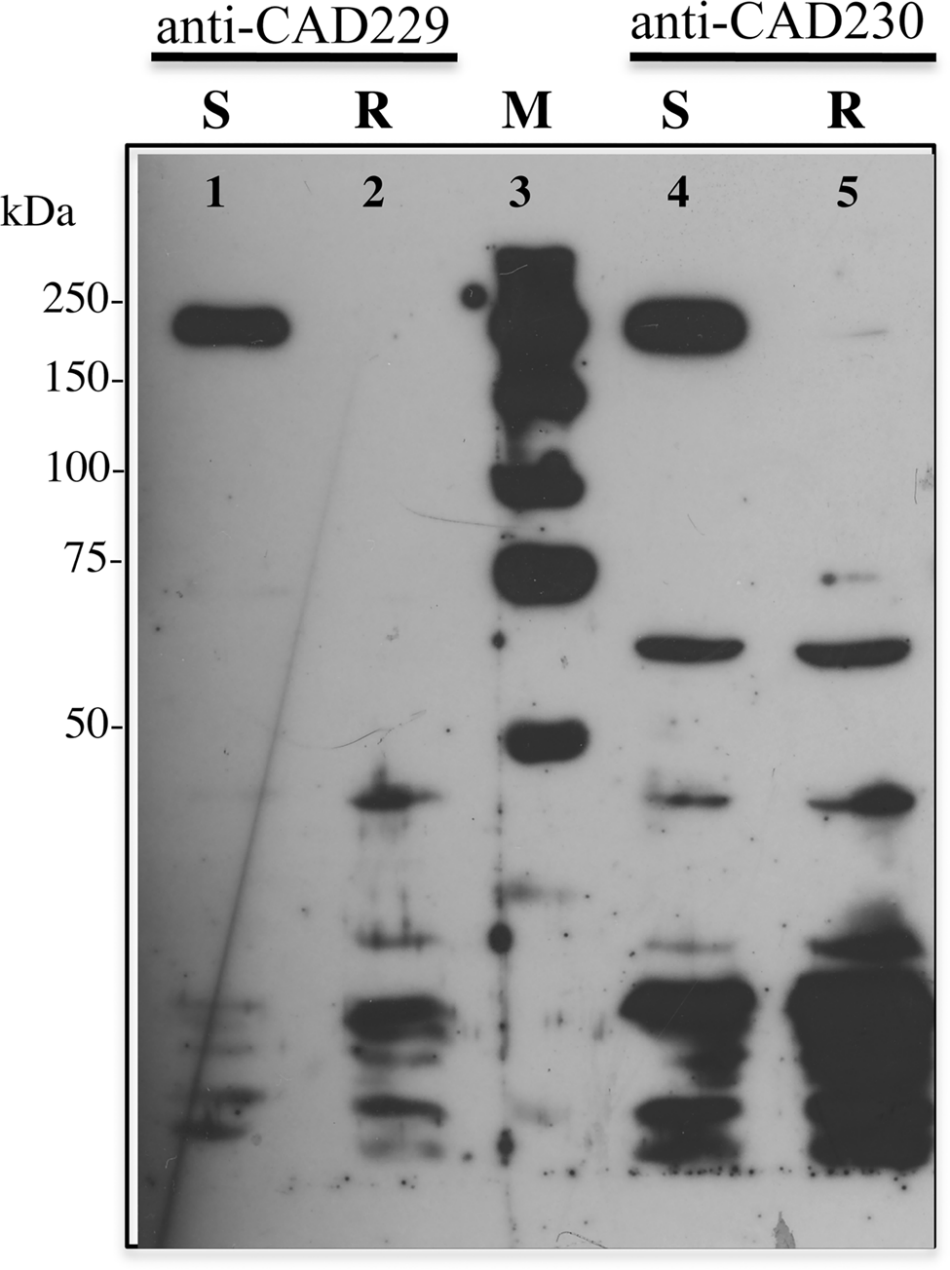
Cadherin detection in resistant and susceptible strains of *Pectinophora gossypiella*. BBMV from susceptible APHIS-S larvae (lanes 1 and 4) and resistant AZP-R larvae (lanes 2 and 5) were separated by SDS-PAGE. We detected cadherin using anti-cadherin antibodies anti-CAD229 (lanes 1 and 2) or anti-CAD230 (lanes 4 and 5). Lane 3 shows the biotinylated molecular weight markers.

In western blots, both anti-cadherin antibodies detected cadherin (ca. 210 kDa) in BBMV from the susceptible APHIS strain, but not in BBMV from the resistant AZP-R strain (Fig 1). Both strains had bands of less than 70 kDa that may be cadherin degradation products (Fig 1).

### Specific Binding of Cry1Ac and Cry1AcMod to BBMV

**Fig 2:**
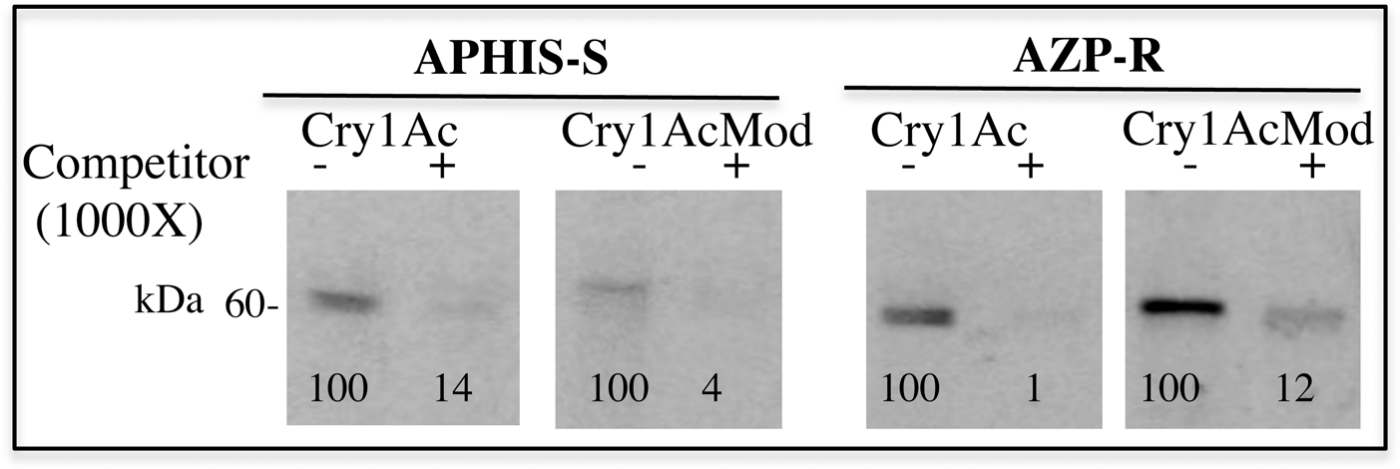
Specific binding of Cry1Ac and Cry1AcMod toxins to BBMV from resistant (AZP-R) and susceptible (APHIS-S) larvae of *P*. *gossypiella*. The left lane of each pair shows total binding with no competitor (-). The right lane shows non-specific binding performed in the presence of 1000-fold excess of unlabeled competitor (Cry1Ac or Cry1AcMod, respectively) (+). The numbers under the bands with competitor (+) represent the optical density of the bands relative to the corresponding bands on the left without competitor (-) (set to 100). Total binding minus non-specific binding equals specific binding.

Specific binding of biotinylated trypsin-activated Cry1Ac and Cry1AcMod to BBMV was similar for APHIS-S and AZP-R (Fig 2). Homologous competition binding assays performed in the presence of 1000-fold excess of unlabeled toxin (unlabeled Cry1Ac added with labeled Cry1Ac or unlabeled Cry1AcMod added with labeled Cry1AcMod) greatly diminished binding of labeled toxin, which indicates it was mostly specific binding (Fig 2).

### Oligomer Formation and Insertion

**Fig 3:**
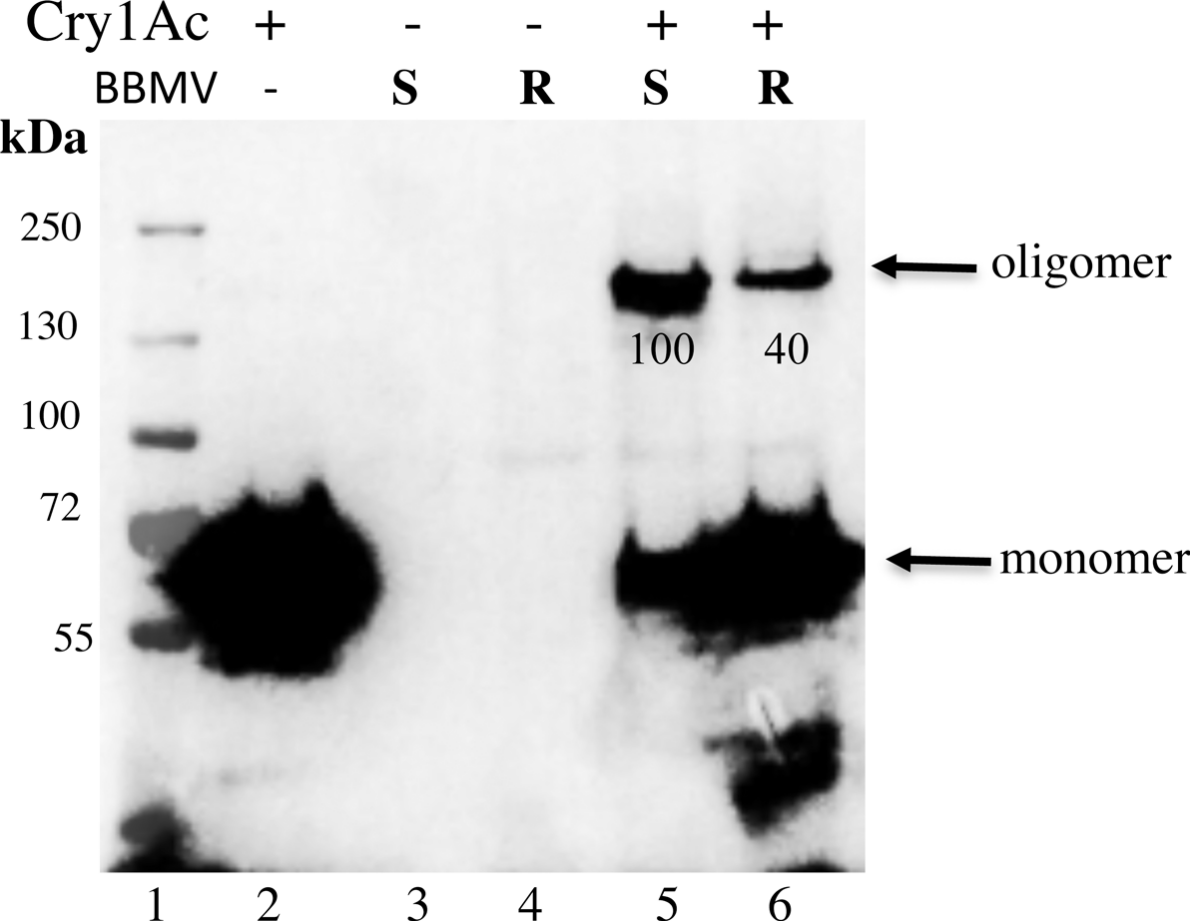
Oligomerization of Cry1Ac in the presence of BBMV from resistant and susceptible larvae of *P*. *gossypiella*. Cry1Ac activated toxin (0.5 μg) was incubated with BBMV (15 μg) from *P*. *gossypiella* susceptible APHIS-S (lane 5), resistant AZP-R (lane 6) separated by SDS-PAGE after three min heating at 50°C and revealed in western blot using anti-Cry1Ac antibody. Lane 1, shows MW markers; lane 2, Cry1Ac activated toxin; lane 3, BBMV from APHIS-S; lane 4, BBMV from AZP-R. Numbers under the 200 kDa bands represent the percentage of pixels relative to the 200 kDa band of APHIS-S BBMV with Cry1Ac toxin that corresponds to 100, calculated after scanning densitometry of the bands.

Oligomers were revealed by western blot using anti-Cry1Ac antibody by loading the samples in SDS-PAGE after heating the samples at 50°C for three min. We focused here on oligomer formation from Cry1Ac incubated with BBMV from resistant and susceptible larvae. We first analyzed oligomerization of Cry1Ac after incubation with BBMV without separation of BBMV by centrifugation. With the optical density of bands from Cry1Ac oligomers (ca. 200 kDa) in BBMV from APHIS-S standardized as 100%, the relative mean optical density of bands from AZP-R was 32% (range = 29 to 40%, paired t-test, t = 3.25, df = 2, P = 0.042, Fig 3 and S1 Table).

**Fig 4:**
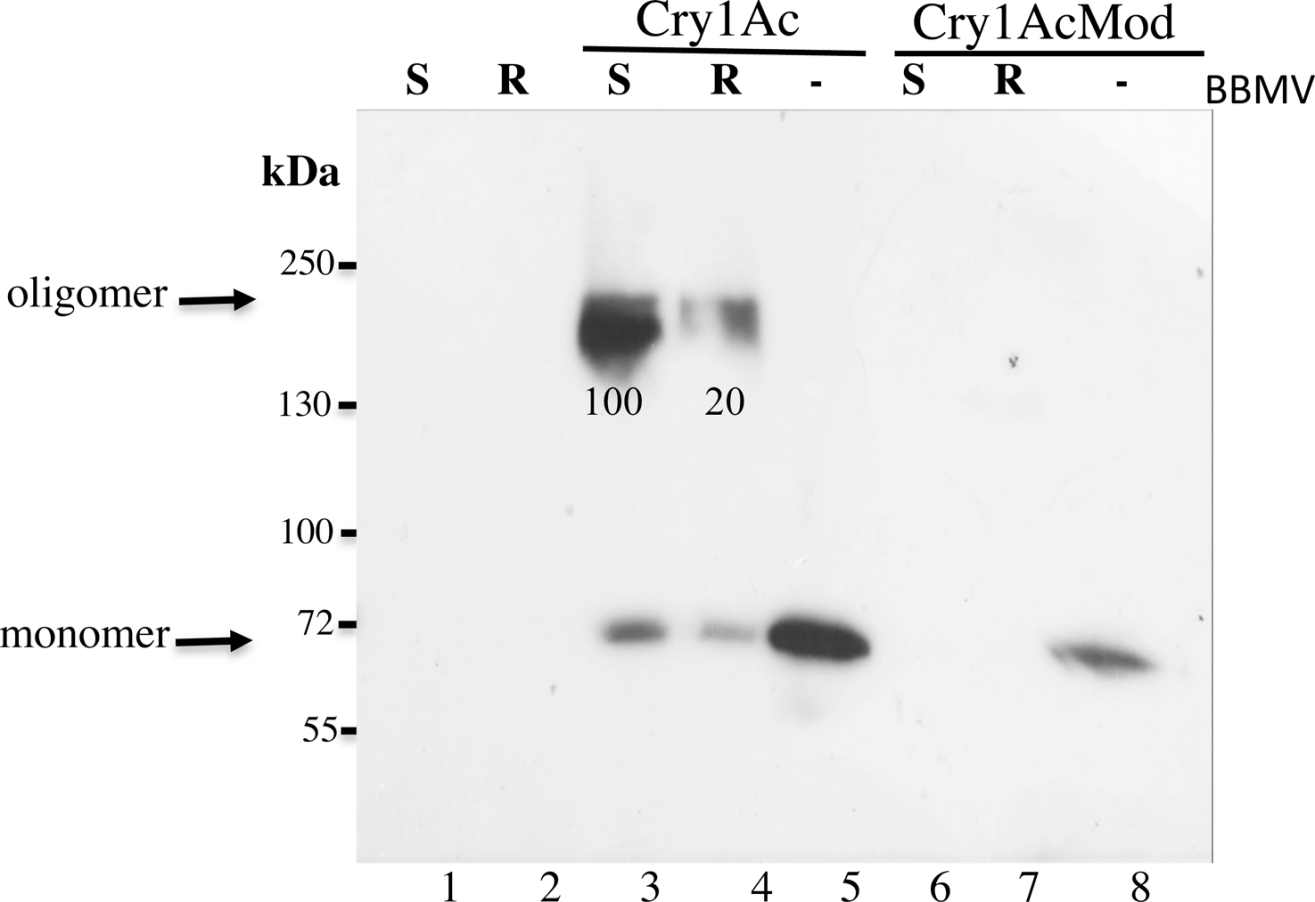
Insertion of oligomers into BBMV from resistant and susceptible larvae of *P*. *gossypiella* with 0.5 μg of Cry1Ac or Cry1AcMod. Cry1Ac or Cry1AcMod activated toxins (0.5 μg) were incubated with BBMV (15 μg) from the different populations and the membrane pellets were recovered by centrifugation, separated by SDS-PAGE after three min heating at 50°C and revealed in western blot assays with anti-Cry1Ac antibody as described in Materials and Methods. Lanes 1 and 2 controls of BBMV without toxin from APHIS-S and AZP-R, respectively; lanes 5 and 8 are controls of soluble Cry1Ac and Cry1AcMod, respectively; lane 3, BBMV from APHIS-S precipitated after incubation with Cry1Ac; lane 4, BBMV from AZP-R precipitated after incubation with Cry1Ac; lane 6, BBMV from APHIS-S precipitated after incubation with Cry1AcMod; lane 7, BBMV from AZP-R precipitated after incubation with Cry1AcMod. Numbers under the 200 kDa bands represent the percentage of pixels relative to the 200 kDa band of APHIS-S BBMV with Cry1Ac toxin that corresponds to 100, calculated after scanning densitometry of the bands.

To analyze insertion of oligomers into the BBMV, we incubated 0.5 μg of activated Cry1Ac or activated Cry1AcMod with 15 μg of BBMV protein, then separated BBMV by centrifugation. With Cry1Ac, relative to BBMV from APHIS-S (standardized as 100%), mean oligomer formation in AZP-R was 22% (range = 9.0 to 30%, paired t-test, t = 15.5, df = 4, P = 0.0001, Fig 4A and S1 Table). With Cry1AcMod, we did not detect oligomers in either strain (Fig 4B).

**Fig 5:**
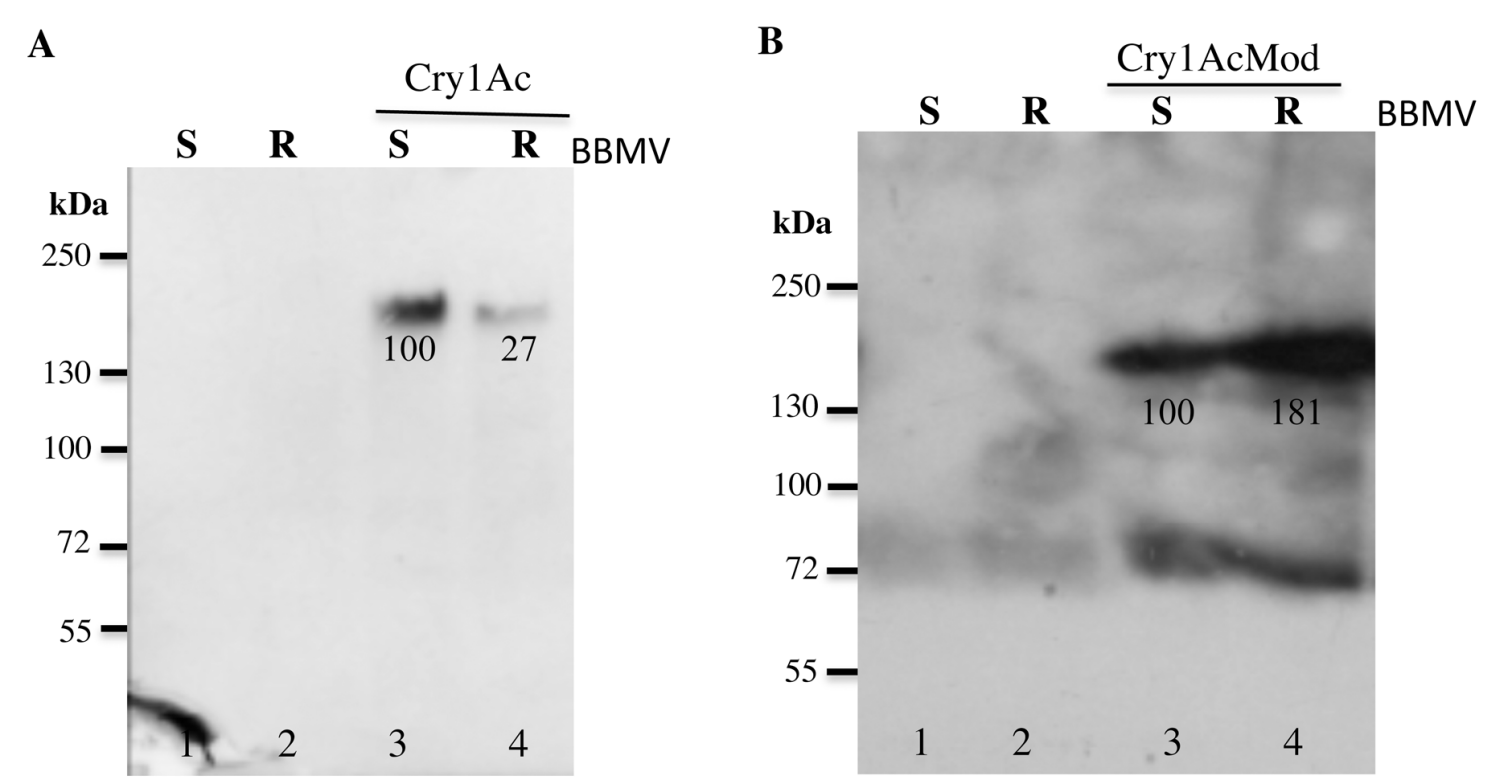
Insertion of oligomers into BBMV from resistant and susceptible larvae of *P*. *gossypiella* with 1.5 μg of Cry1Ac or Cry1AcMod. BBMV (15 μg) from *P*. *gossypiella* were incubated with 1.5 μg of Cry1Ac (Panel A) or Cry1AcMod (Panel B), BBMV were recovered by centrifugation and the membrane pellet was separated by SDS-PAGE after three min heating at 50°C and revealed in western blots using anti-Cry1Ac antibody as described in Materials and Methods. Panel A. lanes 1 and 2 are controls of BBMV without toxin from APHIS-S and AZP-R; lane 3, BBMV from APHIS-S precipitated after incubation with Cry1Ac; lane 4, BBMV from AZP-R precipitated after incubation with Cry1Ac. Panel B. lanes 1 and 2 are controls of BBMV without toxin from APHIS-S and AZP-R; lane 3, APHIS-S precipitated after incubation with Cry1AcMod; lane 4, AZP-R precipitated after incubation with Cry1AcMod. Numbers under the 200 kDa bands represent the percentage of pixels relative to the 200 kDa band of APHIS-S BBMV with Cry1Ac toxin (A) or Cry1AcMod (B) that corresponds to 100, calculated after scanning densitometry of the bands.

Under the same conditions except a three-fold higher concentration (1.5 μg) of activated Cry1Ac, mean oligomer formation for AZP-R relative to APHIS-S was 30% (range = 19 to 46%, paired t-test, t = 7.5, df = 2, P = 0.018, Fig 5A and S1 Table). Under the same conditions but with a three-fold higher concentration (1.5 μg) of activated Cry1AcMod, oligomer formation did not differ significantly between AZP-R and APHIS-S (paired t-test, t = 1.56, df = 2, P = 0.26, Fig 5B and S1 Table). In some replicates we detected small amounts of monomers associated with BBMV (data not shown), which were probably dissembled oligomers because oligomers generated from activated toxin are highly temperature sensitive [23].

## Discussion

Table 1 summarizes the results of this study, including reduced oligomerization of Cry1Ac associated with resistance to Cry1Ac in the AZP-R strain of *P*. *gossypiella*. As far as we know, this is the first study showing an association between resistance and decreased toxin oligomerization. With one notable exception, previous studies of toxin oligomerization have focused on susceptible insects [24, 25]. In the exceptional case, oligomerization of Cry1Ac after incubation with BBMV did not differ between a resistant strain (LF120) and a susceptible strain (96S) of *Helicoverpa armigera* [33].

**Table 1:** Summary of binding and oligomerization of Cry1Ac and Cry1AcMod incubated with BBMV from susceptible and resistant *P*. *gossypiella*.

Insect strain	Toxin					Toxicity [28]
APHIS-S	Cry1Ac	+	++	++	++	+++
AZP-R	Cry1Ac	+	+	+	+	-
APHIS-S	Cry1AcMod	+	NA^a^	-	++	++
AZP-R	Cry1AcMod	+	NA^a^	-	++	++

^a^ NA, data not available; previous results show Cry1AcMod protoxin formed oligomers without BBMV or cadherin [31].

Consistent with previous results showing that resistance to Cry1Ac in AZP-R is tightly linked with deletion mutations disrupting a Cry1Ac-binding cadherin protein [27], the results here reveal reduced expression of full-length cadherin in AZP-R relative to a susceptible strain (Fig 1). By contrast, cadherin expression was similar in the LF120 and 96S strains of *H*. *armigera*, and the cadherin gene sequence differed between these strains by only single amino acid differences [33]. Moreover, the LF120 strain had reduced ALP activity and was derived from the LF60 strain, which harbors a mutation in ABC transporter protein ABCC2 linked with resistance to Cry1Ac [34, 35]. The comparison between the AZP-R strain of *P*. *gossypiella* and the LF120 strain of *H*. *armigera* suggests that the association between resistance and reduced oligomerization depends on the mechanism of resistance.

Consistent with previous results [36], we found that specific binding of Cry1Ac was similar between BBMV from AZP-R and BBMV from the susceptible strain APHIS-S (Fig 2). We hypothesize that these results reflect binding of Cry1Ac to midgut proteins in AZP-R other than cadherin, such as ALP and APN. Given the resistance of AZP-R to Cry1Ac [29, 31], we infer that this binding is not sufficient to confer susceptibility to Cry1Ac.

The fact that oligomerization was reduced in the AZP-R strain but not abolished probably suggests that other insect molecules could also facilitate Cry toxin oligomerization. Also, we did not exclude that the difference in oligomer amounts is due to slower oligomer formation rather than lower amounts being formed. This remains to be determined.

Two different models of oligomerization have been proposed. One of them proposed that oligomerization of Cry toxins follows after monomer insertion into the membrane in contrast to the second model that proposed the formation of a pre-pore oligomeric structure that is responsible for membrane insertion [20]. It is difficult to distinguish between both models. However, the fact that the amount of Cry1Ac monomer is absent or diminish substantially when BBMV were centrifuged and analyzed (Figs 4 and 5), compared with samples where BBMV were not separated by centrifugation (Fig 3) supports that pre-pore oligomers are responsible for membrane insertion. The small amounts of 60 kDa monomer band observed in some samples where BBMV were separated are likely due to disassembled oligomers after temperature treatment since we previously shown that oligomers from activated Cry1Ab monomers are highly sensitive to temperature [23].

The results here indicate that incubation with BBMV from AZP-R yielded oligomer insertion in tests with a high concentration (1.5 μg toxin) of Cry1AcMod, but not Cry1Ac (Fig 5). These new data are consistent with previous findings that Cry1AcMod was much more potent than Cry1Ac against AZP-R [31, 37]. We conclude that the higher efficacy of Cry1AcMod against AZP-R is associated with the increased oligomer formation seen with Cry1AcMod relative to Cry1Ac. However, incubation of Cry1AcMod with BBMV from AZP-R or APHIS-S did not produce oligomer insertion in tests with a lower concentration of Cry1AcMod (0.5 μg toxin) (Figs 2 and 4). In the case of Cry1AbMod, reduced toxicity was associated with reduced efficiency in activated toxin oligomerization [23]. These results could also be related to lower efficiency in oligomerization of Cry1AcMod from activated toxin.

Overall, the results here with Cry1Ac and Cry1AcMod support the conclusion that oligomerization is an important step in the mode of action of these toxins against *P*. *gossypiella*. In addition, the results confirm the role of cadherin in oligomerization of Cry1Ac, but not Cry1AcMod. The previous results from related work with *H*. *armigera* summarized above indicate that reduced oligomerization is not always associated with resistance [33]. More studies comparing oligomerization between resistant and susceptible strains would enable rigorous testing of the hypothesis that decreased oligomerization is associated with disruption of cadherin, but not other mechanisms of resistance.

## Materials and Methods

### Insect Strains

We analyzed the APHIS-S and AZP-R strains of *P*. *gossypiella*. APHIS-S is a susceptible strain that had been reared in the laboratory for more than 30 years without exposure to Bt toxins [38]. AZP-R is a Cry1Ac-resistant strain that was started by pooling survivors of exposure to Cry1Ac in diet from 10 populations derived in 1997 from Arizona cotton fields [29].

### Cry1Ac and Cry1AcMod Toxin Purification

Bt HD73 expressing Cry1Ac or Bt 407 expressing Cry1AcMod [31] were grown at 30°C until complete sporulation (3 to 4 days) in nutrient broth sporulation medium. In the case of the Bt strain producing Cry1AcMod the medium was supplemented with erythromycin at 10 μg ml^-1^. Spores/crystals were washed twice in 300 mM NaCl, 10 mM EDTA. Crystal inclusions were solubilized in an alkaline buffer (50 mM Na_2_CO_3_, 0.2% β-mercaptoethanol, pH 10.5) for 2 h at 37°C. Trypsin activated toxins were obtained by treatment of soluble protoxin with trypsin (TPCK treated trypsin from bovine pancreas, SIGMA Aldrich, St. Louis, MO) in a mass ratio of 1:50 (trypsin: toxin) for 2 h at 37°C after lowering the pH to 8.5 by adding 1:4 (w/w) of 1 M Tris buffer pH 8.5. Phenylmethylsulfonyl fluoride (PMSF) (1 mM final concentration) was added to stop proteolysis. Activated proteins were purified by anion exchange chromatography Mono Q-Sepharose fast flow (GE Healthcare, Little Chalfont, UK) in an AKTA FPLC System (GE Healthcare, Little Chalfont, UK), using a 50 mM Tris-HCl, 50 mM NaCl, pH 8.5 buffer, and a linear NaCl concentration gradient from 50 to 300 mM. Protein concentrations were determined by the method of Bradford, using bovine serum albumin as a standard.

### Midgut Brush Border Membrane Vesicles (BBMV) Purification

*P*. *gossypiella* midgut tissues from third instar larvae were dissected and stored immediately at -70°C. BBMV were prepared by the magnesium precipitation method as described by Wolfersberger et al 1987 [39] and stored at -70°C until used. The BBMV protein concentrations were determined with the Lowry DC protein assay (BioRad, Hercules, CA) using bovine serum albumin as a standard.

### Binding of Cry1Ac and Cry1AcMod to BBMV

To determine the effect of cadherin mutations in *P*. *gossypiella* AZP-R on Cry1Ac and Cry1AcMod binding, we performed binding analysis of the activated toxins to BBMV. The crystal inclusions of Cry1Ac, and Cry1AcMod were solubilized, activated with trypsin and labeled with biotin. The total binding of biotinylated proteins after incubation with BBMV isolated from each susceptible and resistant insect was analyzed in absence of competitor. The non-specific binding was analyzed in homologous competition experiments after incubation of these toxins with the BBMV in the presence of 1000-fold excess of the corresponding unlabeled toxin. Trypsin activated monomeric toxins were labeled with biotinyl-*N-*hydroxy-succinimide ester according to the manufacturer’s instructions (Amersham Biosciences). Non-specific binding was determined by measuring binding of 5 nM labeled toxin in the presence of 1000-fold molar excess of unlabeled toxin after 100 min. Proteins were incubated at 25°C with 10 μg BBMV protein in 100 μl binding buffer (PBS, 0.1% BSA, 0.1%Tween 20, pH 7.6). After incubation the unbound toxin was removed by centrifugation for 10 min at 14,000 x*g*. The pellet containing BBMV and bound toxin was washed twice with 100 μl binding buffer, suspended in 10 μl of PBS pH 7.6, and 10 μl sample loading buffer 2X (0.125 mM Tris-HCl, pH 6.8, 4% SDS, 20% glycerol, 10% 2-mercaptoethanol, and 0.01% bromophenol blue). Samples were boiled 3 min, loaded in 10% SDS-PAGE gels and electrotransferred to nitrocellulose membranes. Bound labeled toxin was identified by incubating with streptavidin-peroxidase conjugate (Millipore) (1:20000 dilution) for 1h, developing with luminol (Santa Cruz Biotechnology Inc.). The optical density of the 60 kDa bands was measured by using ImageJ program (http://imagej.nih.gov/ij/). All binding assays were performed in triplicate.

#### Specific binding

Binding assays of Cry1Ac or Cry1AcMod biotinylated toxins were performed as previously described [23]. Total binding was measured in binding assays after 100 min of incubating 10 μg BBMV protein with 5 nM labeled toxin in 100 μl binding buffer. For competition analysis 1000-fold excess of unlabeled toxin was added at the same time as labeled toxin. All assays were performed in triplicate. Numbers under the bands in Fig 2 represent the percentage of each band on the blot calculated after scanning optical density of the bands and using the 60 kDa band of the same size in the gel with the highest optical density as 100% reference.

### Identification of Cadherin Protein

Western blots were performed to identify cadherin protein. Cadherin in *P*. *gossypiella* was identified with two different antibodies raised against two fragments of cadherin from *P*. *gossypiella* the CR8-CR9 fragment (antibody 229) and CR8-CR11 fragment (antibody 230) [27]. BBMV protein (10 μg) from each insect were boiled 3 min in sample loading buffer, loaded into 10% SDS-PAGE and electrotransferred to a nitrocellulose membrane, blocked with skimmed milk (5%), detected with anti-cadherin polyclonal antibodies (1/30,000; 1 h) and visualized with a goat anti-rabbit antibody coupled with horseradish peroxidase (Sigma, St. Louis, MO) (1/5000; 1 h), followed by SuperSignal chemiluminescent substrate (Pierce, Rockford, Il) as described by the manufacturers.

### Toxin Oligomerization in BBMV

Oligomerization of Cry1Ac or Cry1AcMod toxins in BBMV was analyzed as previously described [21]. Activated toxins were used at two different concentrations (0.5 or 1.5 μg), these proteins were incubated with 15 μg of BBMV protein for 1 h at 37°C in a total volume of 50 μl of alkaline buffer, pH 10.5. Control samples contained only BBMV. The reactions were stopped with 1 mM PMSF and the BBMV were recovered by 30 min centrifugation at 50,000 rpm at 4°C or directly analyzed without centrifugation. The pellet was washed once with 100 μl of alkaline buffer, and finally suspended in 50 μl of the same buffer. Laemmli sample buffer 4X was added and incubated three min at 50°C. After heating, samples were separated in 8% SDS-PAGE, electro transferred to PVDF membrane and revealed in western blot assays as described above using anti-Cry1Ac antibody (1/30,000; 1 h) as the primary antibody. All assay were performed in triplicate

## Supporting Information

S1 Table Scanning densitometry data for comparing oligomer formation in resistant (AZP-R) and susceptible (APHIS-S) strains of *P*. *gossypiella*. (DOCX)

## Abbreviations

Cry: Crystal δ-endotoxin
Bt: *Bacillus thuringiensis*
3d-Cry: three-domain-Cry toxins
LC_50_: mean lethal concentration
BBMV: brush border membrane vesicles
ALP: alkaline phosphatase
APN: aminopeptidase-N
