# Supplementary material for: Binding and Oligomerization of Modified and Native Bt Toxins in Resistant and Susceptible Pink Bollworm
Source: PLoS One. 2015 Dec 3;10(12):e0144086. doi: 10.1371/journal.pone.0144086 (PMC4669222; doi:10.1371/journal.pone.0144086)
Supplement: S1 Table — (DOCX) [file pone.0144086.s001.docx]

Table S1. Scanning densitometry data for comparing oligomer formation in resistant (AZP-R) and susceptible (APHIS-S) strains of *P. gossypiella*

| Fig. 3 Oligomerization of Cry1Ac in the presence of BBMV | | | | |
| --- | --- | --- | --- | --- |
|  | Optical density | | | |
| Replicate | APHIS-S | AZP-R | (AZP-R/APHIS-S) X 100% | |
| 1 | 19854 | 7942 | 40.0 % |  |
| 2 | 61097 | 19552 | 32.0 % |  |
| 3 | 55129 | 15987 | 29.0 % |  |
| Mean | 45360 | 14494 | 32.0 % |  |
| Std dev | 22289 | 5947 | 5.7 % |  |

| Fig. 4 Insertion of oligomers into BBMV (0.5 μg Cry1Ac) | | | | |
| --- | --- | --- | --- | --- |
|  | Optical density | | | |
| Replicate | APHIS-S | AZP-R | (AZP-R/APHIS-S) X 100% | |
| 1 | 65100 | 16306 | 25.0 % |  |
| 2 | 76092 | 15218 | 20.0 % |  |
| 3 | 46142 | 41040 | 9.0 % |  |
| 4 | 70352 | 21056 | 29.9 % |  |
| 5 | 59852 | 14301 | 23.9 % |  |
| Mean | 63508 | 14204 | 21.6 % |  |
| Std dev | 11431 | 6199 | 7.9 % |  |

| Fig. 5A Insertion of oligomers into BBMV (1.5 μg Cry1Ac) | | | | |
| --- | --- | --- | --- | --- |
|  | Optical density | | | |
| Replicate | APHIS-S | AZP-R | (AZP-R/APHIS-S) X 100% | |
| 1 | 82609 | 15701 | 19.0 % |  |
| 2 | 62259 | 16086 | 25.8 % |  |
| 3 | 81398 | 37054 | 45.5 % |  |
| Mean | 75422 | 22947 | 30.1 % |  |
| Std dev | 11416 | 12219 | 13.8 % |  |

| Fig. 5B Insertion of oligomers into BBMV (1.5 μg Cry1AcMod) | | | | |
| --- | --- | --- | --- | --- |
|  | Optical density | | | |
| Replicate | APHIS-S | AZP-R | (AZP-R/APHIS-S) X 100% | |
| 1 | 41389 | 74912 | 181.0 % |  |
| 2 | 66081 | 63436 | 96.0 % |  |
| 3 | 51540 | 69842 | 135.5 % |  |
| Mean | 53003 | 69397 | 137.5 % |  |
| Std dev | 12411 | 5751 | 42.5 % |  |
